# Investigation on the proton range uncertainty with spectral CT‐based virtual monoenergetic images

**DOI:** 10.1002/acm2.14062

**Published:** 2023-06-13

**Authors:** Libing Zhu, Yi Du, Yahui Peng, Xincheng Xiang, Xiangang Wang

**Affiliations:** ^1^ Institute of Nuclear and New Energy Technology Tsinghua University Beijing PR China; ^2^ Key Laboratory of Carcinogenesis and Translational Research (Ministry of Education/Beijing), Department of Radiotherapy Peking University Cancer Hospital & Institute Beijing PR China; ^3^ School of Electronic and Information Engineering Beijing Jiaotong University Beijing PR China

**Keywords:** optimal energy pairs, range uncertainty, spectral CT, virtual monoenergetic imaging

## Abstract

**Objective:**

The stopping power ratio (SPR) prediction error will contribute to the range uncertainty of proton therapy. Spectral CT is promising in reducing the uncertainty in SPR estimation. The purpose of this research is to determine the optimal energy pairs of SPR prediction for each tissue and to evaluate the dose distribution and range difference between the spectral CT with the optimal energy pairs method and the single energy CT (SECT) method.

**Methods:**

A new method was proposed based on image segmentation to calculate the proton dose with spectral CT images for the head and body phantom. CT number of each organ region were converted to SPR with the optimal energy pairs of each organ. The CT images were segmented into different organ parts with thresholding method. Virtual monoenergetic (VM) images from 70 keV to 140 keV were investigated to determine the optimal energy pairs for each organ based on Gammex 1467 phantom. The beam data of Shanghai Advanced Proton Therapy facility (SAPT) was employed in matRad (an open‐source software for radiation treatment planning) for the dose calculation.

**Results:**

The optimal energy pairs were obtained for each tissue. The dose distribution of two tumor sites (brain and lung) were calculated with the aforementioned optimal energy pairs. The maximum dose deviation between spectral CT and SECT at the target region was 2.57% and 0.84% for the lung tumor and brain tumor respectively. The range difference between spectral and SECT was significant with 1.8411 mm for the lung tumor. γ passing rate was 85.95% and 95.49% for the lung tumor and brain tumor with the criterion 2%/2 mm.

**Conclusions:**

This work presents a way to determine the optimal energy pairs for each organ and to calculate the dose distribution based on the more accurate SPR prediction.

## 1 INTRODUCTION

Proton therapy is sensitive to the uncertainties due to the high‐dose‐gradient feature.^1^ One significant uncertainty is the stopping power ratio (SPR) prediction related range uncertainty. Conventionally, the CT number to SPR conversion curve is obtained with single energy CT (SECT). Schaffner and Pedroni^2^ claimed that the uncertainty of SPR prediction with SECT was ±1.1% for soft tissue and ±1.8% for bone, contributing to 1−3 mm proton range uncertainty. Dual energy CT has been widely investigated to mitigate the SPR related range uncertainty.^3^, ^4^, ^5^, ^6^

Recently, spectral CT attracts broad interest in reducing the SPR estimation errors. There are several techniques to realize spectral CT imaging, for example, dual‐layer detector spectral CT,^7^, ^8^, ^9^ rapid kV switching technology, dual source scanner, etc. The GE revolution scanner used a rapid kV switching technology to achieve dual energy CT scan and generate virtual monoenergetic (VM) CT images. Different ways of SPR calibration can be done through DECT, that is, dual energy CT stoichiometric calibration, using pairs of VM images, performing standard SECT stoichiometric calibration on VM images. Je^10^ calculated the optimal energy for proton SPR estimation using the VM images. The optimal energy was 160 keV based on the composite range uncertainty. The residuals of CT‐to‐SPR conversion curve for different tissue was energy‐dependent. Näsmark et al.^11^ investigated the optimal energy pairs with dual‐energy CT‐generated VM images for the SPR prediction of different tissues. SPR prediction was based on the formalism of Jackson and Hawkes.^12^ The optimal energy pairs of SPR prediction was 52/53 keV, 48/51 keV, and 84/85 keV for lung group, soft tissue group and bone group, respectively.^11^ Therefore, it is significant to employ the CT‐to‐SPR conversion curves of different energies for different tissues. The proton dose calculation based on pairs of VM images of optimal energy pairs has not been investigated. In this paper, we employ the optimal energy pairs to convert the CT number to SPR for each tissue and calculate the proton dose. The beam range is compared between the spectral CT and SECT calculations.

## 2 MATERIAL AND METHODS

### 2.1 Spectral CT image acquisition

A Gammex 1467 phantom (Gammex, Inc.) and a pediatric phantom were scanned with GE Revolution CT (GE Healthcare Waukesha) with scanning parameters in Table 1. The VM images were acquired using Gemstone Spectral Imaging (GSI) from 70 keV to 140 keV. The CT‐to‐SPR conversion curves were obtained based on different VM images.

**TABLE 1:** Scanning parameters and reconstruction parameters of the spectral CT.

Scanning parameters	Pediatric phantom	Gammex 1467 phantom
Slice thickness (mm)	2.5	5
Source to detector distance (mm)	1097.61	1097.61
Source to patient distance (mm)	625.61	625.61
Exposure time (s)	250	250
X‐ray tube current (mA)	200	320
Convolution kernel	Standard	Standard
Pitch (mm)	0.5781	0.8613

In order to compare with the traditional stoichiometric calibration method, 120 kVp images of the phantoms were obtained with GE Revolution CT for both phantoms.

### 2.2 Optimal energy pairs determination with VM images

**FIGURE 1:**
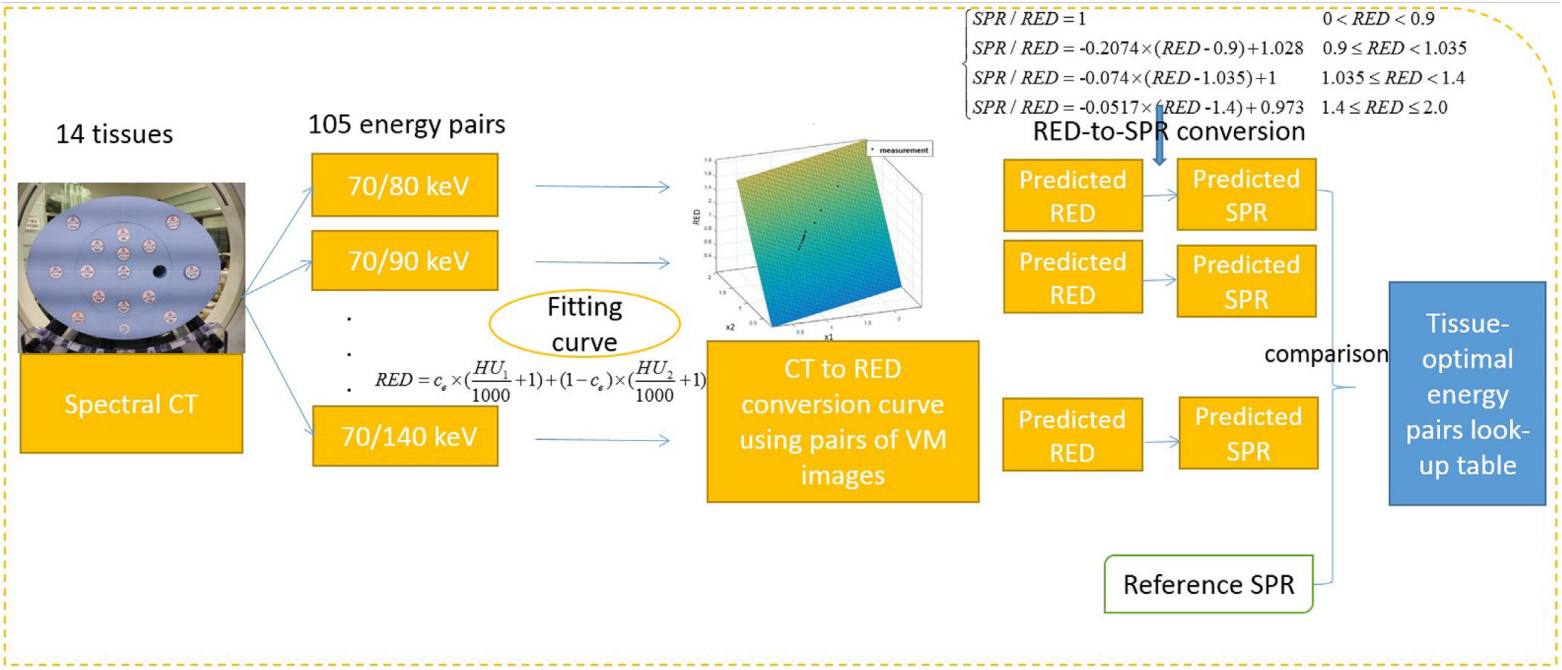
CT‐to‐SPR conversion process using pairs of VM images to determine the optimal energy pairs of each tissue.

VM images of the Gammex 1467 phantom were generated in 5 keV step between 70 and 140 keV. Figure 1 demonstrates the CT‐to‐SPR conversion process using pairs of VM images. In total, 105 energy pairs were investigated for the optimal energy pairs determination of different tissues. Fourteen tissue inserts were investigated to establish a look‐up table for different tissues. Reference SPR of each tissue were calculated with Bethe's stopping power formula. Equation (1)^13^ was employed to calibrate relative electron density (RED) with pairs of VM images. After obtaining the parameter *c_e_* in Equation (1) by the fitting, the predicted RED of each tissue was calculated using pairs of VM images. Kanematsu investigated a polyline conversion function from RED to SPR^14^ shown in Equation (2). With the polyline conversion function and the predicted RED, the predicted SPRs of each tissues were calculated. The SPR residuals of different energy pairs was calculated as a function of energy pairs. The residuals denote the deviation between the predicted SPR and the reference values. The determined optimal energy pairs is the one with the minimized residual. Therefore, a tissue‐to‐optimal energy pairs look‐up table was established with Gammex 1467 phantom.

(1) $$RED=c_{e}\times \left(\frac{HU_{1}}{1000}+1\right)+\left(1-c_{e}\right)\times \left(\frac{HU_{2}}{1000}+1\right)$$

(2) $$\left\{\begin{array}{cc} SPR/RED=1 & 0<RED<0.9\\ SPR/RED=-0.2074\times (RED-0.9)+1.028 & 0.9\leq RED<1.035\\ SPR/RED=-0.074\times (RED-1.035)+1 & 1.035\leq RED<1.4\\ SPR/RED=-0.0517\times (RED-1.4)+0.973 & 1.4\leq RED\leq 2.0 \end{array}\right.$$

where *c_e_* is a constant to be fitted with the reference RED and CT‐values. *HU_1_* and *HU_2_* denote the CT value of two VM images.

### 2.3 Organ segmentation‐based dose calculation for SAPT

Shanghai Advanced Proton Therapy facility (SAPT) is a synchrotron‐based facility with 94 energies. The proton dose was calculated with matRad (an open‐source software for radiation treatment planning of intensity‐modulated photon, proton, and carbon ion therapy).^15^ The beam data (integral depth dose, spot size and virtual source‐to‐axis distance) was employed in matRad for the beam modelling.

**FIGURE 2:**
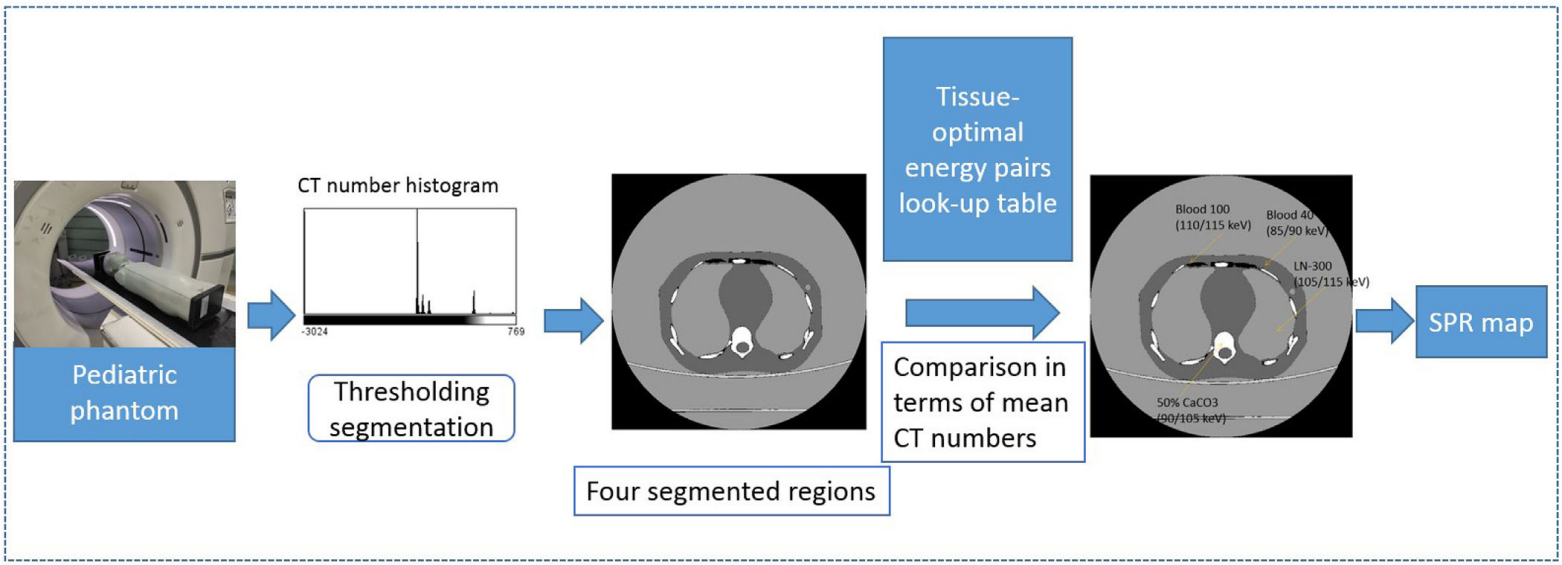
Scheme of the SPR calculation based on the segmented spectral CT images.

Figure 2 illustrates the dose calculation with the predicted SPR maps using pairs of VM images. When calculating the proton dose with the pediatric phantom, the CT images were firstly segmented into four regions, named Region 1, Region 2, Region 3, Region 4, with thresholding method. By comparing the mean CT numbers of four segmented regions with that of the Gammex 1467 tissue inserts, it is found that CT number of four regions of pediatric phantom is approximately same to that of 50% CaCO_3_ (HU = 881.44), HE Blood 100 (HU = 106.44), HE Blood 40 (45.61), and LN‐300 (−702.66) lung insert of Gammex 1467 phantom. With the CT‐to‐optimal energy pairs look‐up table described in Section 2.2, each segmented region's optimal energy pairs is determined. Therefore, at each segmented region, its optimal energy pairs were employed to convert the CT number to SPR using the Equations (1) and (2). Compared with the traditional method where all the tissues were converted to SPR with one pair of energy, our proposed method converts the CT number to SPR with four energy pairs. The calculated SPR was then imported into matRad for the dose calculation. The number of fractions is 30 with the total dose 60 Gy. The beam angles were −45^o^ and 45° for the lung tumor while −45^o^ and −90^o^ beam was employed for brain tumor. The couch angles were 0^o^. A constant RBE of 1.1 was utilized for the optimization. The planning target volume (PTV) was spheroidal. When calculating the proton dose based on the spectral CT and SECT, all the computation settings were the same except the SPR maps.

## 3 RESULTS

### 3.1 SPR prediction for each tissue

**FIGURE 3:**
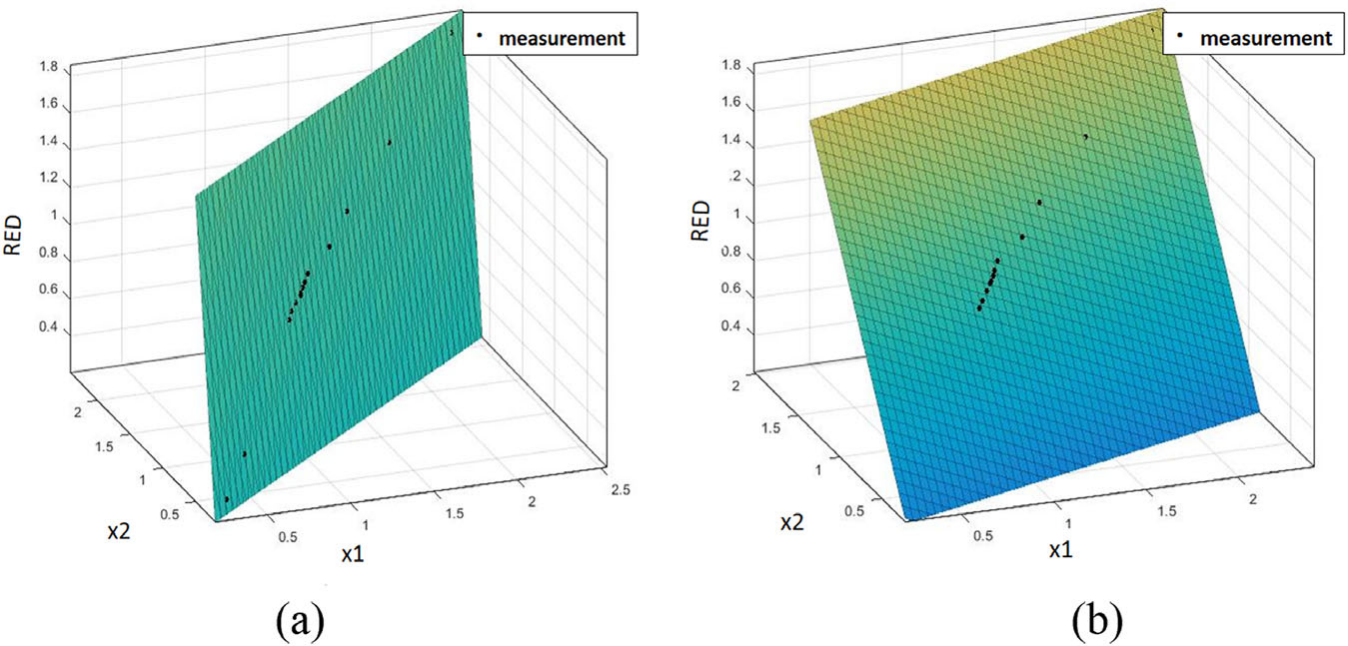
CT‐to‐RED conversion curve using pairs of VM images: (a) 70/75 keV and (b) 70/105 keV (×1 = 1+HU_1_/1000, ×2 = 1+HU_2_/1000).

The CT number to RED conversion curves are obtained using pairs of VM images (Figure 3). Fourteen conversion curves were investigated while only four conversion curves were employed for the dose calculation because the pediatric phantom was segmented into four regions. The predicted SPR is then calculated with Equation (2) to convert RED to SPR. Region of interest (ROI) with the same size are chosen to determine the CT number of each tissue insert.

**FIGURE 4:**
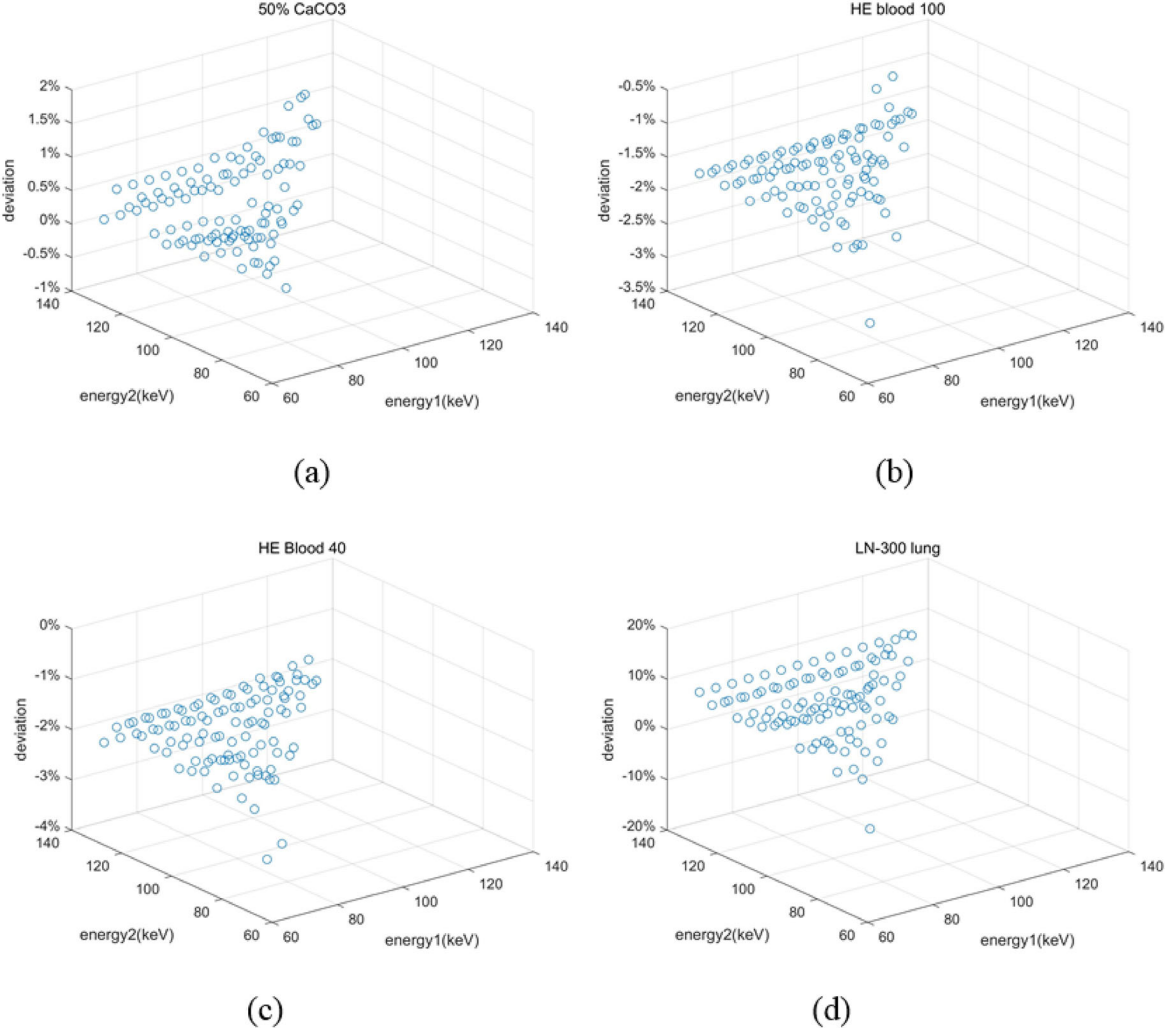
SPR deviation between the predicted SPR and reference value as a function of energy pairs for (a) 50% CaCO3, (b) HE Blood 100, (c) HE Blood 40, and (d) LN‐300 lung.

SPR residuals of 14 tissue surrogate inserts are calculated as a function of energy pairs. In Figure 4, SPR residuals of 50% CaCO_3_, HE blood 100/40 and LN‐300 lung are illustrated as a function of energy pairs. Z axis is the SPR deviation between the predicted SPR based on pairs of VM images and the reference value. It is found that the deviation is energy pairs‐dependent. An optimal energy pairs can be determined with the minimized residuals.

For each tissue, the SPR prediction of 105 energy pairs is analyzed. The reference REDs are taken from vendor's manual and employed for reference SPR calculation with Bethe Block equations. The predicted SPRs are computed for 14 different tissues of 105 energy pairs. The minimum SPR difference between the prediction and reference values is shown in Table 2. The deviation is the discrepancy between the predicted SPRs and the reference values with the SECT method. By using spectral CT, the fitting residuals of 30% CaCO_3_ can be reduced from −0.94% to 0.01%. The predicted SPR residuals are mitigated to 0.02%, 2.09%, and −0.04% for adipose, LN‐450 lung and LN‐300 lung respectively. The 14 tissue inserts can be divided into three groups: bone group (HE cortical bone, 50% CaCO_3_, 50% CaCO_3_, HE Inner Bone), soft tissue group (HE blood 100, 70, 40, Liver, Brain, solid water, Breast 50:50, general adipose) and lung tissue group (LN‐450, LN‐300). Root mean square error (RMSE) is 0.22%, 0.75%, and 0.92% for bone group, soft tissue group and lung group respectively based on pairs of VM images. RMSE is 1.57%, 2.26%, and 1.55% for bone group, soft tissue group and lung group based on SECT. RMSE is reduced with pairs of VM images compared with that of SECT. In conclusion, spectral CT outperforms SECT for predicting SPR in terms of RMSE. For each tissue, the corresponding CT to SPR conversion curve is utilized for patient dose calculation.

**TABLE 2:** SPR prediction of spectral CT and SECT for Gammex 1467 phantom (the tissue‐optimal energy pairs look‐up table).

		Spectral CT			SECT	
Tissue surrogate	Reference SPR	Predicted SPR	Minimum SPR difference	Optimal energy pair/kev	Predicted SPR	deviation
HE Cortical Bone	1.6970	1.6977	0.05%	75/80	1.6983	0.08%
50% CaCO_3_	1.4161	1.4160	−0.01%	90/105	1.4179	0.13%
30% CaCO_3_	1.2479	1.2481	0.01%	110/120	1.2362	−0.94%
HE Inner Bone	1.1493	1.1472	−0.18%	90/95	1.1595	0.89%
HE blood 100	1.0947	1.0894	−0.48%	110/115	1.0966	0.18%
HE Blood 70	1.0672	1.0629	−0.40%	95/100	1.0705	0.31%
HE Liver	1.0488	1.0471	−0.17%	95/100	1.0508	0.18%
HE Blood 40	1.0311	1.0289	−0.21%	85/90	1.0394	0.81%
HE Brain	1.0232	1.0238	0.06%	75/80	1.0297	0.63%
HE solid water	1.0073	1.0085	0.13%	105/115	1.0067	−0.06%
HE Breast 50:50	0.9831	0.9830	−0.01%	105/115	0.9714	−1.19%
HE general adipose	0.9585	0.9587	0.02%	95/105	0.9429	−1.63%
LN‐450 lung	0.4400	0.4492	2.09%	100/105	0.4541	3.20%
LN‐300 lung	0.2800	0.2799	−0.04%	105/115	0.2736	−2.29%

### 3.2 Proton dose calculation for lung tumor and brain tumor

**FIGURE 5:**
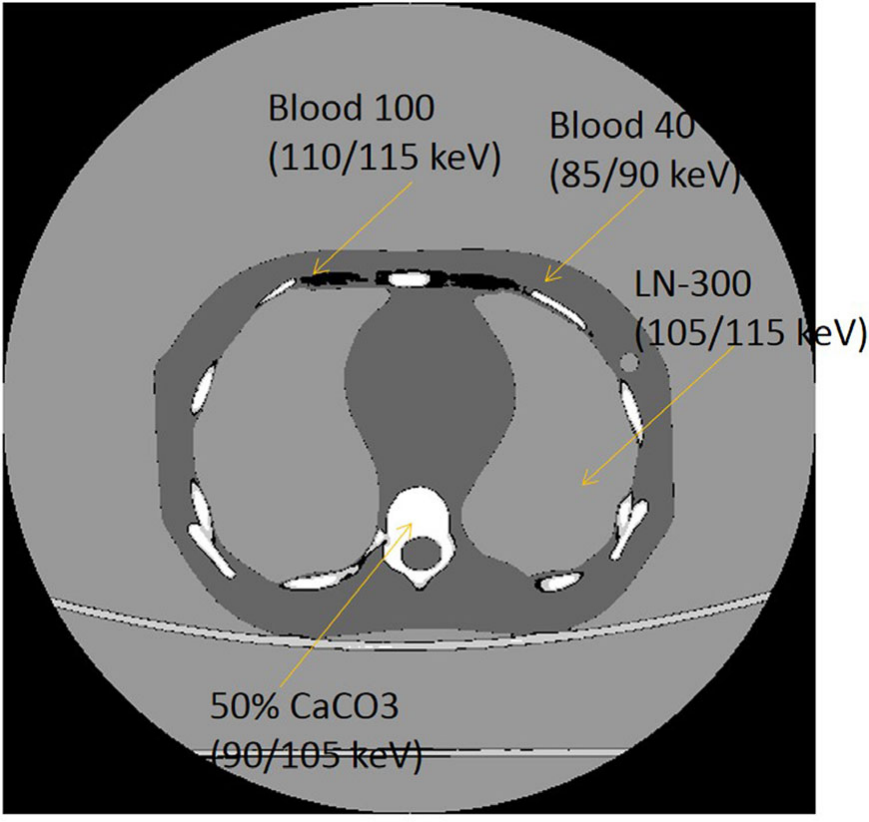
Segmentation of the pediatric phantom into four regions: 50% CaCO3, Blood 100, Blood 40, LN‐300.

**FIGURE 6:**
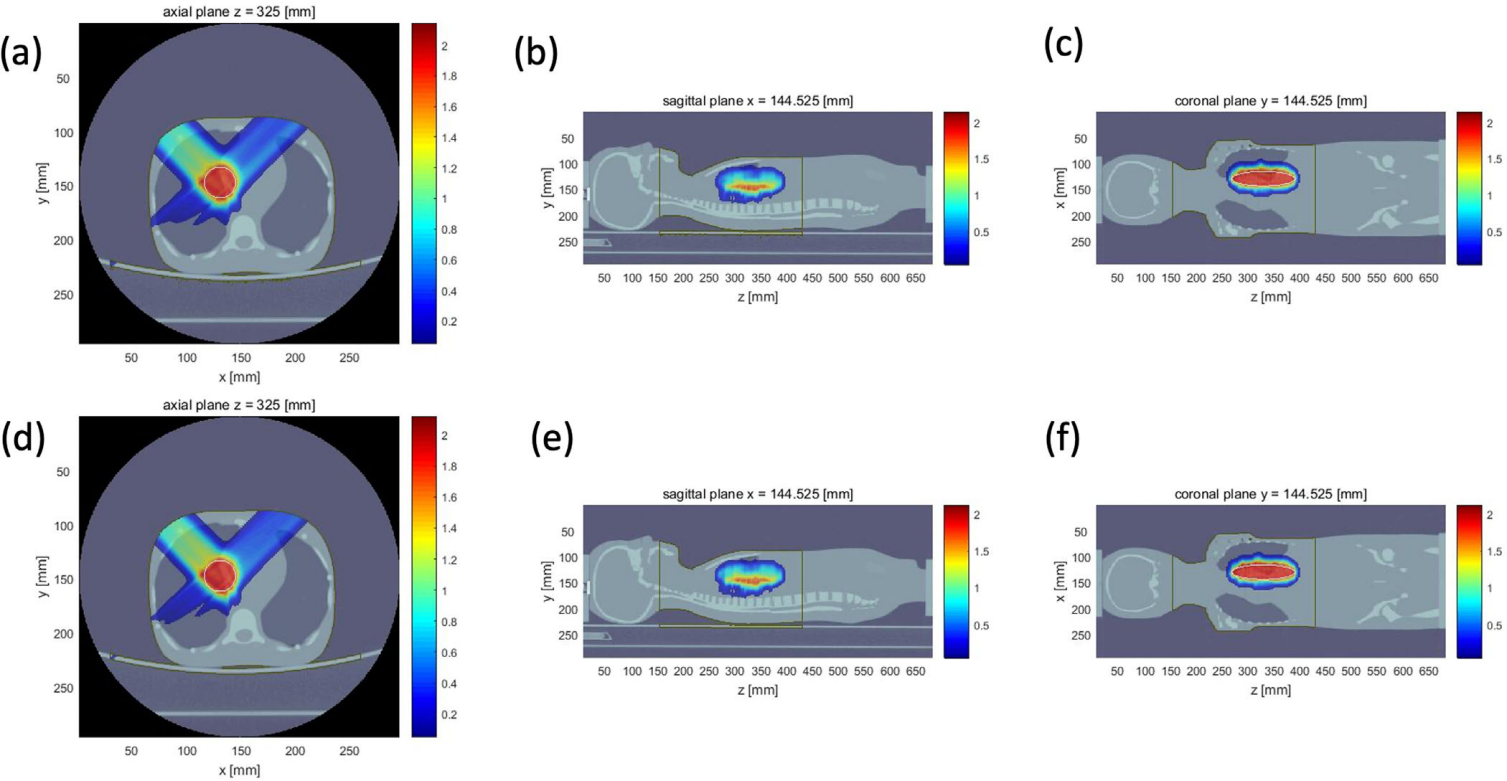
(a)‐(c) Spectral CT based lung tumor dose calculation at axial plan, coronal plane and sagittal plane, (d)‐(f) SECT based lung tumor dose calculation at axial plan coronal plane and sagittal plane.

Compared with the traditional method where all the tissues were converted to SPR with one pair of energy, our proposed method converts the CT number to SPR with four energy pairs. The calculated SPR was then imported into matRad for the dose calculation. The segmented region and its determined optimal energy pairs was shown in Figure 5. The optimal energy pairs was determined for each region by comparing the mean CT number with the tissue‐optimal energy pairs look‐up table (Table 2) in terms of the closest CT number. We calculate the dose distribution of lung tumor with spectral CT and SECT (Figure 6) based on the body phantom. Significant dose and range difference are found between the spectral CT and SECT calculations.

**FIGURE 7:**
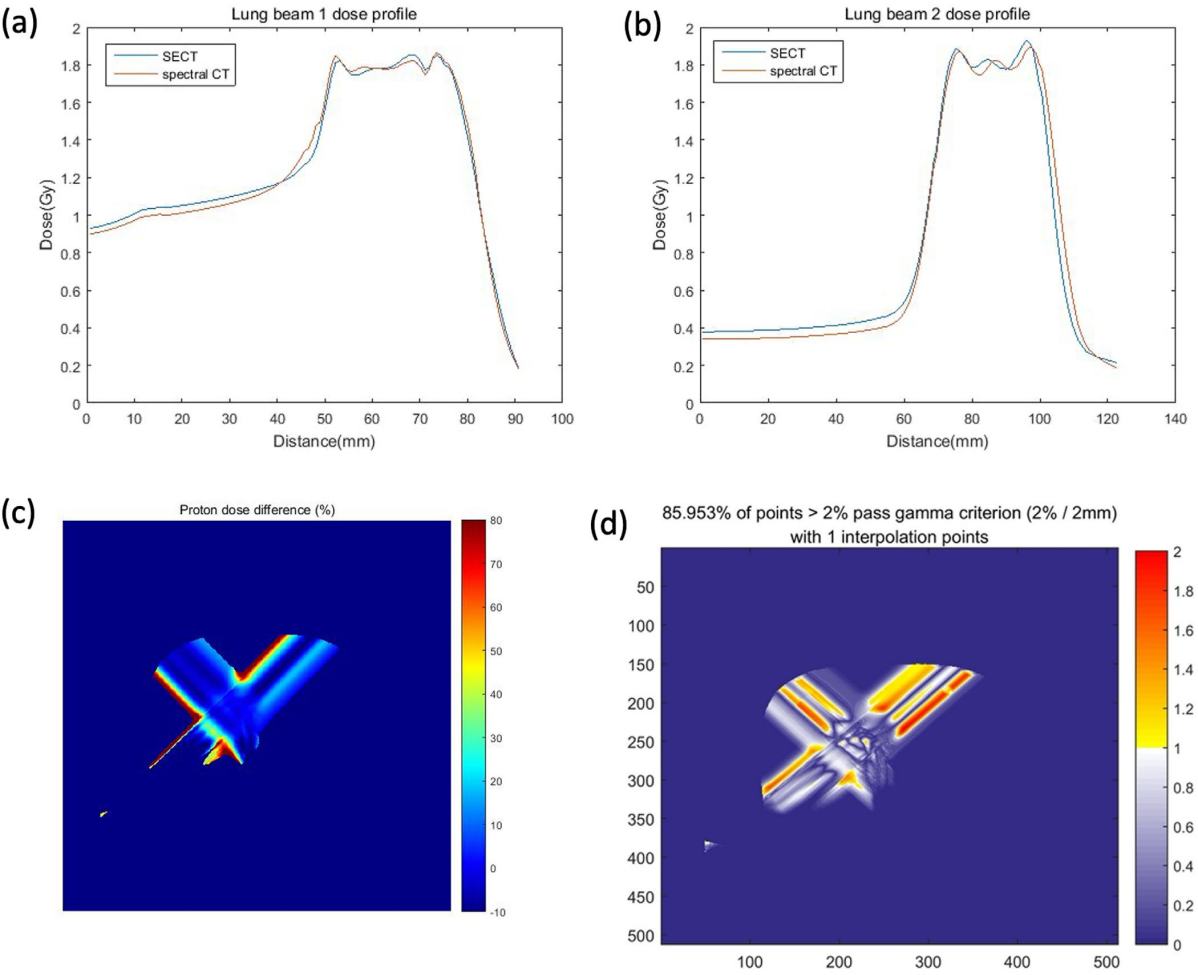
Dose profile of SECT and spectral CT (a) along beam −45° (b) along beam 45° (c) Relative percent dose deviation between spectral CT and SECT for lung tumor (d) γ map with criterion 2%/2 mm.

Figure 7a,b demonstrate the dose profile along the beam central axis. Relative dose difference between spectral CT and SECT is calculated (Figure 7c). Maximum dose deviation at entrance region and targeted region is 77.94% and 2.57%, respectively, for the beam with angle −45^o^. The great dose deviation at entrance region can be attributed to that the lung SPR prediction deviation is reduced from −2.29% to −0.04% with spectral CT. 3D γ passing rate of two fields is 85.95% for the criterion 2%/2 mm (Figure 7d). The prominent dose difference is attributed to the SPR prediction of lung between spectral CT and SECT.

**FIGURE 8:**
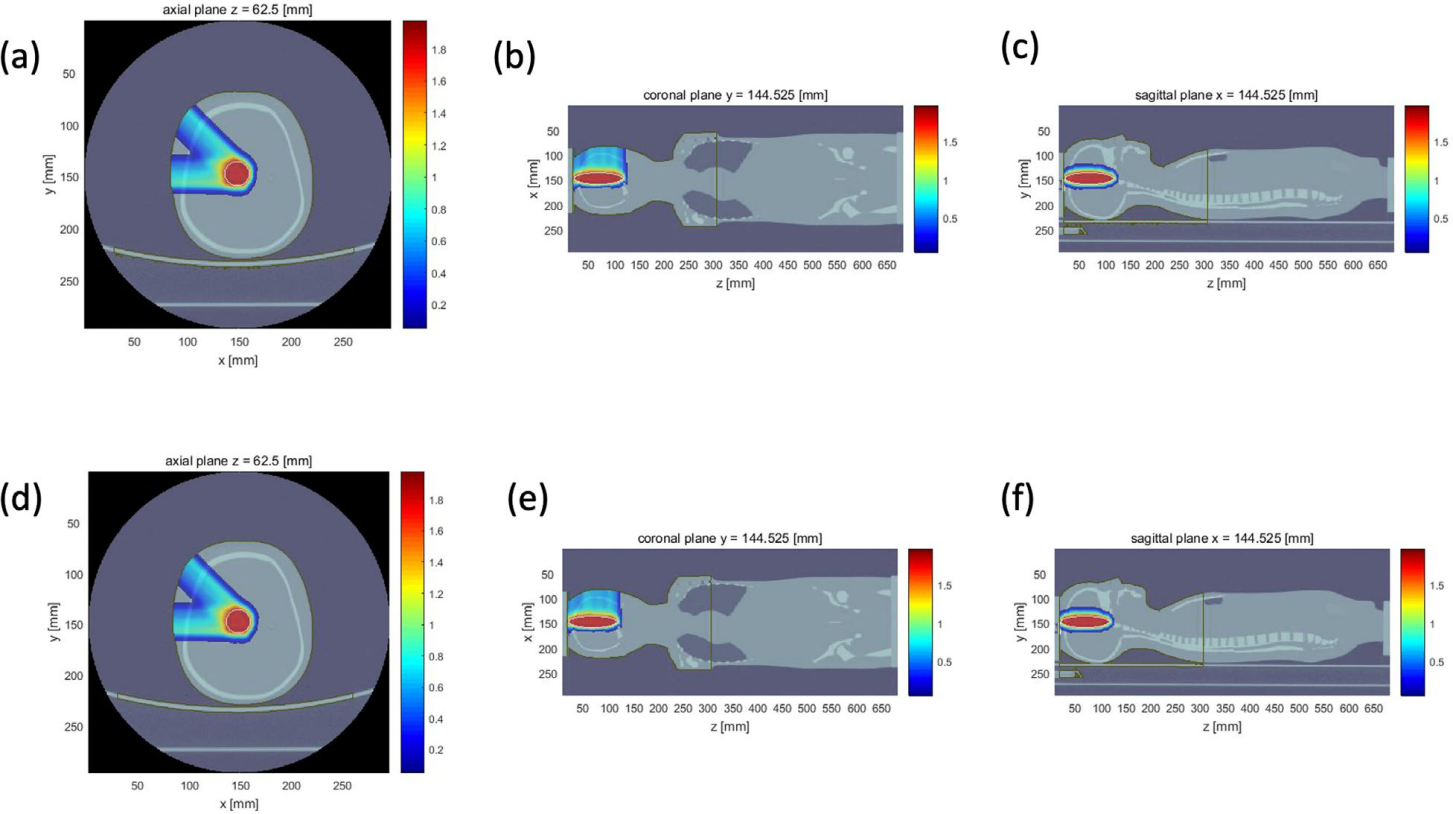
(a)‐(c) Spectral CT based brain tumor dose calculation at axial plan, coronal plane and sagittal plane, (d)‐(f) SECT based brain tumor dose calculation at axial plan coronal plane and sagittal plane.

**FIGURE 9:**
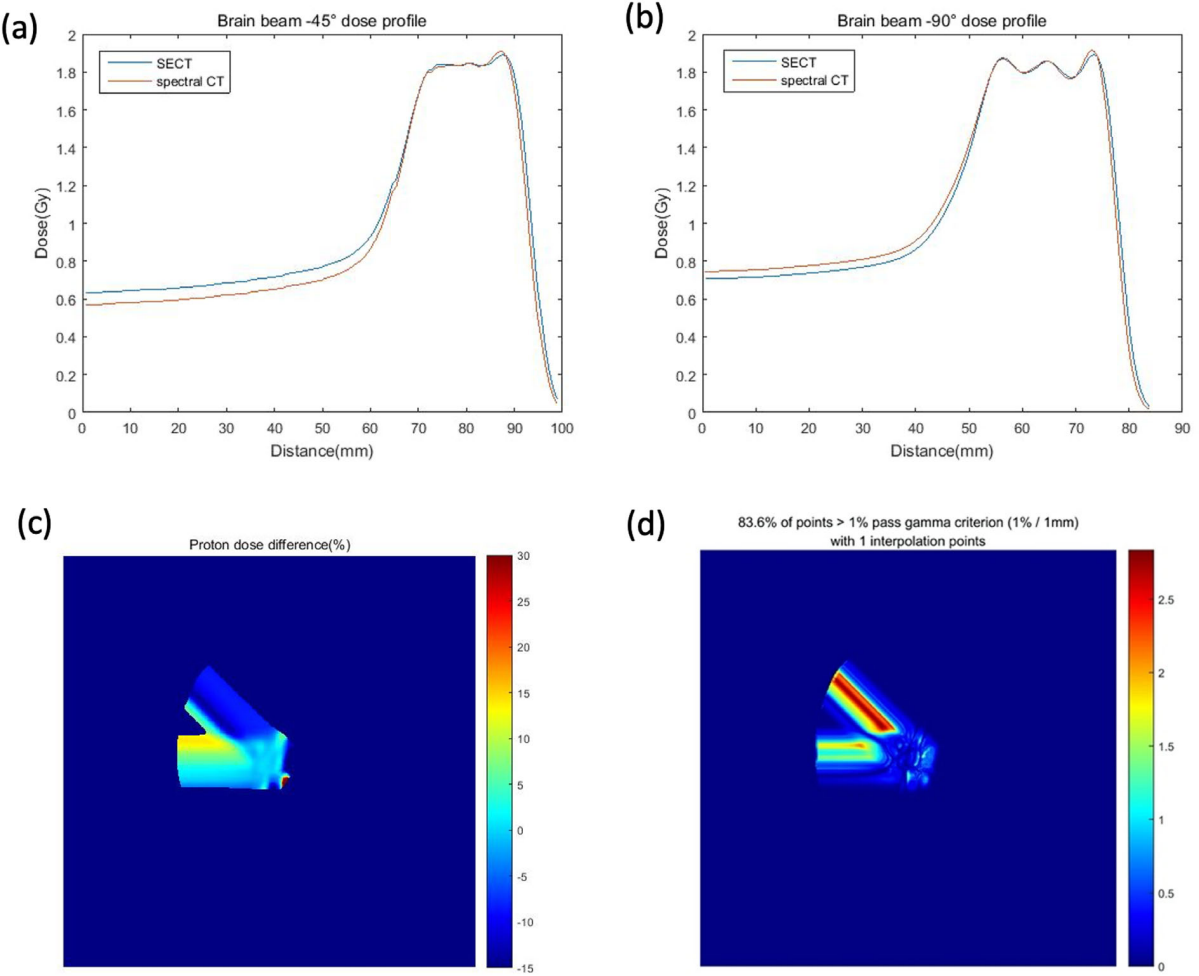
Dose profile of SECT and spectral CT (a) along beam −45° (b) along −90° (c) Relative percent dose deviation between spectral CT and SECT for brain tumor (d) γ map with tolerance 1%/1 mm.

Figure 8 demonstrates the dose distribution of brain tumor based on spectral CT and SECT. The dose profiles along the beam central axis are plotted with the depths (Figure 9). The beam angles are −45° and −90°.

The maximum dose discrepancy between spectral CT and SECT is 13.8% and 0.84% at the entrance region and the targeted region respectively for the beam with angle −90^o^. 3D γ passing rate of the two fields is 95.49% and 83.6% with the criterion 2%/2 mm and 1%/1 mm, respectively.

### 3.3 Proton beam range comparison

The proton range was calculated along the beam direction for the lung tumor and brain tumor (Table 3). The central axis profile was plotted to calculate the range for each field. The range difference between spectral and SECT of beam 45° is significant with 1.8411 mm for lung tumor. The range deviation between spectral CT and SECT is small for brain tumor, 0.69 mm and 0.61 mm for the beam −45° and −90°, respectively. SPR prediction deviation of the soft tissue could contribute to the significant dose deviation at the entrance region.

**TABLE 3:** Proton beam range comparison between spectral CT and SECT.

Tumor site	Beam direction	Range (90% distal dose)/mm		range deviation	range difference/mm
		Spectral CT	SECT		
Lung tumor	Beam −45°	78.02	77.79	0.31%	0.24
	Beam 45°	101.28	99.44	1.85%	1.84
Brain tumor	Beam −45°	89.90	90.58	‐0.76%	‐0.69
	Beam −90°	75.11	75.72	‐0.80%	‐0.61

## 4 DISCUSSION

In this paper, the optimal energy pairs with spectral CT is determined by comparing the predicted SPR of 14 tissue surrogates with the reference value. The SPR estimation errors of 11 tissues are below 0.5%. The minimum SPR residuals is 2.09% for LN‐450 lung with spectral CT. With SECT, the SPR residual for the tissue is 3.20%. An improvement is made with spectral CT for LN‐450 lung.

Näsmark^11^ investigated the dependency on imaging and scanning parameters. This paper is focused on the dose and range comparison between spectral CT and SECT. In Näsmark's research, the tissues were divided into three tissue groups: lung group, soft tissue group and bone group to determine the optimal energy pairs. Root‐mean‐square error (RMSE) was 7.2%, 0.5%, and 0.4% for lung tissue group, soft tissue group and bone group respectively. In our research, RMSE is 0.11%, 0.27%, and 0.65% for bone group, soft tissue group and lung tissue group respectively because we employed each individual tissue's optimal energy pairs. Je^10^ investigated the imaging, modelling and inherent uncertainties for different tissue groups and selected 160 keV as the optimal energy. However, our objective is to calculate the dose distribution based on each tissue's optimal energy pairs.

In Table 2, the spectral CT calibrations were outperformed by SECT for Blood 100/70 and solid water inserts. The generated VM images are in 5 keV step ranging from 70 to 140 keV. Thus, the investigated energy pairs are limited. Further research should be focused on the smaller energy step with more comprehensive calculation using the pairs of VM images. The 14 tissue inserts can be divided into three groups: bone group (HE cortical bone, 50% CaCO3, 50% CaCO3, HE Inner Bone), soft tissue group (HE blood 100, 70, 40, Liver, Brain, solid water, Breast 50:50, general adipose) and lung tissue group (LN‐450, LN‐300). Root mean square error (RMSE) is 0.22%, 0.75%, and 0.92% for bone group, soft tissue group and lung group respectively based on pairs of VM images. RMSE is 1.57%, 2.26%, and 1.55% for bone group, soft tissue group and lung group based on SECT. RMSE is reduced with pairs of VM images compared with that of SECT. The maximum dose deviation between spectral CT and SECT at target region is 2.57% and 0.84% for lung tumor and brain tumor respectively. The dose discrepancy is significant for lung tumor because the lung SPR prediction is more accurate with spectral CT with smaller SPR residuals.

Considering the complex tissue composition of real patients, a generalized optimal pairs for different tissue groups (lung group, soft tissue group and bone group) can be employed. RMSE of each group is reduced with the pairs of VM images compared with that of SECT. In a clinical scenario it is relatively easy to determine whether a pixel contains lung tissue, soft tissue, or bone. It does not matter if the pixel contains breast tissue, muscle, or adipose tissue, as the generalized soft tissue energy pair will yield good results for all of them. Thus, the real patient images can be divided into three groups and can mitigate the error of thresholding method because the CT number varies significantly at different group. The high Z metal heterogeneities can cause uncertainty in image segmentation. Proper artefacts correction method should be imposed before the image segmentation. The proton dose calculation is based on the analytical algorithm. The algorithm was compared against with the clinical treatment planning system Syngo (Siemens, Erlangen, Germany) by the developers.^15^ Good agreement was observed with global gamma‐analysis pass rates ≥99.67% (acceptance criteria 2%/2 mm). In addition, the open source software can support Monte Carlo (MC) dose calculation and we are investigating the validation of the MC calculation.

## 5 CONCLUSION

This work presents a way to determine the optimal energy pairs for each organ and to calculate the dose distribution based on the more accurate SPR prediction. The optimal energy pairs for each tissue were determined by comparing the SPR estimation of spectral CT with the reference value. When calculating the patient dose distribution, the images were segmented into different tissues and each tissue were converted to SPR with its optimal energy pairs. Comparing with SECT method, there are smaller residuals of the CT‐to‐SPR conversion curve with the spectral CT method. Further study will be focused on the proton dose calculation of the clinical patients.

## AUTHOR CONTRIBUTIONS

Conceptualization, Libing Zhu, Yi Du, and Xiangang Wang; methodology, Libing Zhu, Yi Du, Yahui Peng, Xiangang Wang, and Xincheng Xiang; data acquisition and analysis, Libing Zhu, Yi Du, and Xincheng Xiang; interpretation, Libing Zhu, Yi Du, and Xiangang Wang; writing—original draft preparation, Libing Zhu, Yahui Peng, and Xiangang Wang; writing—review and editing, Xincheng Xiang, Yi Du, Yahui Peng, and Xincheng Xiang; supervision, Yahui Peng, Xincheng Xiang, and Xiangang Wang

## CONFLICT OF INTEREST

The authors declare no conflicts of interest.
